# Infiltration-Assisted Mechanical Strengthening of 3D-Printed Polypropylene Lattice and Thin-Walled Tube Structures

**DOI:** 10.3390/polym17192604

**Published:** 2025-09-26

**Authors:** Hakkı Özer

**Affiliations:** Department of Automotive Engineering, Faculty of Engineering, Bursa Uludag University, Bursa 16059, Turkey; hakkiozer@uludag.edu.tr

**Keywords:** lattice structures, thin-walled structures, polypropylene, epoxy infiltration, energy absorption, 3D-printed polymer structures, mechanical strengthening

## Abstract

This study presents a viscosity-controlled epoxy infiltration strategy to mitigate common production defects, such as interlayer bond weaknesses, step gaps, and surface roughness, in 3D-printed polypropylene lattice and tube structures. To address these issues, epoxy resin infiltration was applied at four distinct viscosity levels. The infiltration process, facilitated by ultrasonic assistance, improved epoxy penetration into the internal structure. The results indicate that this method effectively reduced micro-voids and surface irregularities. Variations in epoxy viscosity significantly influenced the final internal porosity and the thickness of the epoxy film formed on the surface. These structural changes directly affected the energy absorption (EA) and specific energy absorption (SEA) of the specimens. While performance was enhanced across all viscosity levels, the medium-viscosity specimens (L-V2 and L-V3), with a mass uptake of ~37%, yielded the most favorable outcome, achieving high SEA (0.84 J/g) and EA (252 J) values. This improvement was mainly attributed to the epoxy filling internal voids and defects. Mechanical test results were further supported by SEM observations and validated through statistical correlation analyses. This work constitutes one of the first comprehensive studies to employ epoxy infiltration for defect mitigation in 3D-printed polypropylene structures. The proposed method offers a promising pathway to enhance the performance of lightweight, impact-resistant 3D-printed structures for advanced engineering applications.

## 1. Introduction

Thin-walled tubes are essential engineering components contributing to passive vehicle safety. During a collision, they protect drivers and passengers by dissipating kinetic energy through plastic deformation [1,2,3]. These structures are also attractive due to their low weight [4], ease of fabrication, and low production costs [5,6], and are therefore widely employed in the automotive [7,8,9], aerospace [10] and defense industries [11,12].

Comparative analyses of thin-walled structures with varying cell numbers have shown that multi-cell configurations can increase energy absorption capacity while maintaining mass efficiency [13,14,15]. Ma et al. [16] investigated the dynamic energy absorption behavior of thin-walled, cellular sandwich circular tubes and reported that, in polypropylene (PP) structures, increasing the number of cells while reducing wall thickness enhanced energy absorption without increasing mass. Similarly, Faraz et al. [17] demonstrated that fully PP sandwich tubes exhibited higher efficiency than aluminum counterparts under impact loading, providing lower peak forces and higher specific energy absorption (SEA). Although PP exhibits high mechanical performance, particularly under low-speed impact, its application in this context remains limited. Further investigation into their axial impact behavior is therefore warranted.

The demand for lightweight vehicles has driven the development of more complex and efficient structural designs. Thin-walled tubes with a polymer matrix, manufactured via 3D printing, enable intricate geometries that provide both high SEA and low mass [18]. Previous studies have examined the impact resistance of 3D-printed polymer materials through experimental and numerical methods, demonstrating their potential for energy absorption applications [13,19]. Multi-cell thin-walled tubes produced by additive manufacturing have been reported to exhibit superior energy absorption compared to those manufactured using conventional techniques [20,21].

Advancements in 3D printing technologies have also facilitated the fabrication of crashworthy elements in lattice as well as tubular configurations [22,23,24]. Lattice structures can recover their original geometry after unloading due to their elastic deformation capability and exhibit high fatigue resistance under repeated loading. These properties have led to their widespread use in diverse engineering applications [25,26]. Lattice structures, owing to their high specific strength, controlled deformation, and energy absorption efficiency, are considered effective design options for impact and crash scenarios [27,28]. Geometrically, multi-cell architectures distribute loads over larger surfaces, reducing local stress concentrations [29]. Load sharing among cell walls helps maintain structural rigidity while minimizing weight, enabling the simultaneous achievement of lightness and high load-bearing capacity. The mechanical performance of lattice structures with different cell types and connection configurations has been extensively investigated under both static and dynamic conditions [25,30,31].

Among various configurations, body-centered cubic (BCC) and face-centered cubic (FCC) lattice architectures exhibit superior energy absorption potential despite their low densities [29,32,33]. AlMahri et al. [25] produced three lattice configurations—BCC, FCC, and BCC+FCC hybrids—at different relative densities and examined their impact resistance and SEA under quasi-static and dynamic loading. Comparative tests on specimens produced using fused deposition modeling (FDM) with ABS and Tough-PLA filaments showed that FCC structures, in particular, achieved higher plateau stress and SEA values. Likewise, Song et al. [34] investigated 3D-printed lattice structures with various geometries for energy absorption and found that FCC architectures demonstrated the most favorable mechanical performance. Their polymer specimens, manufactured via FDM, were tested under quasi-static axial compression.

Polypropylene is a widely used thermoplastic in engineering applications due to its low density, high chemical resistance, impact strength [35], and recyclability [36]. However, extrusion-based additive manufacturing of PP suffers from severe warpage and insufficient layer-to-layer bonding due to shrinkage and low surface energy, even when process parameters are optimized [37,38,39,40]. Similar limitations of semicrystalline PP in ME-AM have also been reported in composite systems; for instance, the incorporation of microcrystalline cellulose fillers improved reinforcement to some extent, but issues such as shrinkage, interfacial debonding, and dimensional instability remained significant [41]. Consequently, mechanical characterization studies of PP-based 3D-printed structures are scarce [42]. Although PP exhibits considerable deformation capacity under low-speed impact, its potential for impact energy absorption has not been comprehensively explored. Similar limitations are also reported for other engineering polymers fabricated via additive manufacturing. For example, thin-walled polymer tubes tend to buckle under axial loads, with early failure occurring at weak points caused by manufacturing defects. Previous studies have shown that such weaknesses can be mitigated by incorporating filler materials into the cells or tube interiors, thereby increasing the critical buckling load [43,44,45,46]. Wang et al. enhanced buckling resistance without compromising mass efficiency by filling polymer composite cylinders with foam [47]. Previous approaches, such as ultrasonic vibration treatment, improved adhesion to some extent [48], but did not fully resolve the interlayer bonding limitations. A recent study also explored reinforcement strategies for additively manufactured composites [49]; however, a systematic evaluation of viscosity-controlled infiltration in semi-crystalline PP has not been reported.

In this study, viscosity-controlled epoxy infiltration was applied to mechanically reinforce PP-based lattice and tube structures produced via 3D printing. We conducted ultrasonic bath-assisted infiltration at four viscosities ranging from 150 to 1070 cP, and examined structural performance in relation to the viscosity–morphology–mechanical integrity interplay. This study shows that infiltration viscosity governs the balance between pore filling and surface film formation, which in turn governs crashworthiness parameters (SEA, EA, and MCF). By combining morphological observations, statistical correlation analyses, and mechanical testing, we introduce a production–structure–performance framework for PP-based 3D-printed structures. The findings provide practical guidance for lightweight, impact-resistant designs, including crashworthy automotive tubes and aerospace lattice components.

## 2. Materials and Methods

### 2.1. Materials

Test specimens in this study were produced using BASF polypropylene (PP) filament (1.75 mm diameter, BASF SE, Ludwigshafen, Germany) The filament was used directly in the FDM-based manufacturing process without pre-drying. The PP material had a density of 0.90 g/cm^3^ and a melting temperature of 141 °C.

The infiltration process used Resin Slim Extra, a two-component epoxy system (Marker Kimya, Mersin, Turkey). The resin and hardener were mixed at a weight ratio of 100:50, according to the manufacturer’s specifications. The viscosity values were measured under controlled environmental conditions and were found to be temperature-dependent, as summarized in Table 1: 1000–1070 cP at 15–17 °C, 500–560 cP at 23–25 °C, 300–350 cP at 28–30 °C, and 150–180 cP at 34–36 °C. These values were critical for determining the resin’s ability to penetrate the porous structure. Viscosity was determined using a calibrated rotational viscometer, and each target level was obtained by adjusting the resin temperature in the ultrasonic bath (ISOLAB Laborgeräte GmbH, Wertheim, Germany), ensuring repeatable conditions.

### 2.2. Sample Fabrication

#### 2.2.1. Geometrical Design of Lattice and Tube Structures

**Figure 1:**
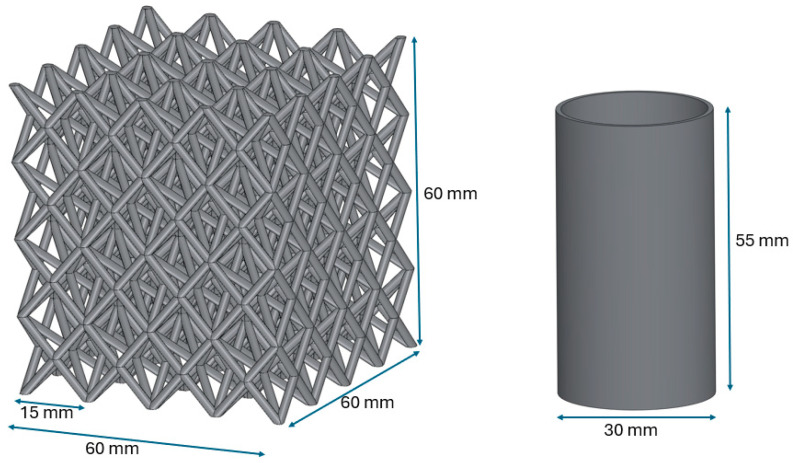
CAD models of lattice and tube structures.

The mechanical performance of both cage and tube structures was compared in order to evaluate the effectiveness of epoxy infiltration. All geometries were designed using SolidWorks software (version 2018, Dassault Systèmes, Vélizy-Villacoublay, France). The cage design combined Face-Centered Cubic (FCC) and Body-Centered Cubic (BCC) unit cells, both known for high specific strength and energy dissipation. The hybrid structure consisted of four unit cells in each spatial direction, with overall dimensions of 60 × 60 × 60 mm^3^. The cross-sectional thickness of the lattice struts was 2 mm. The cylindrical tubes had an outer diameter of 30 mm, a height of 55 mm, and a wall thickness of 2 mm. The simple geometry of the tubes minimized internal deformation caused by thermal stresses during 3D printing, serving as a reliable reference for comparison with the cage structures. For both lattice and tube specimens, printing parameters such as layer height and speed were optimized to ensure maximum dimensional stability. The CAD models of both geometries are shown in Figure 1.

#### 2.2.2. Additive Manufacturing Parameters

All samples were fabricated using an Ekser Plus FDM-type 3D printer (Ermaksan, Bursa, Turkey) equipped with a 0.4 mm nozzle. Printing was conducted with the layers oriented perpendicular to the loading direction (along the Z-axis) to emphasize the influence of interlayer bonding during mechanical testing. All process parameters, including print speed, layer height, and extrusion temperature, were kept constant, as detailed in Table 1. Although 100% infill was selected in the slicing software (Cura 2024), micro-voids were detected in the inner regions due to PP’s high shrinkage tendency and weak interlayer adhesion. These defects adversely affected mechanical strength. To mitigate these defects and strengthen interlayer bonding, epoxy infiltration was applied.

#### 2.2.3. Epoxy Infiltration Procedure

**Figure 2:**
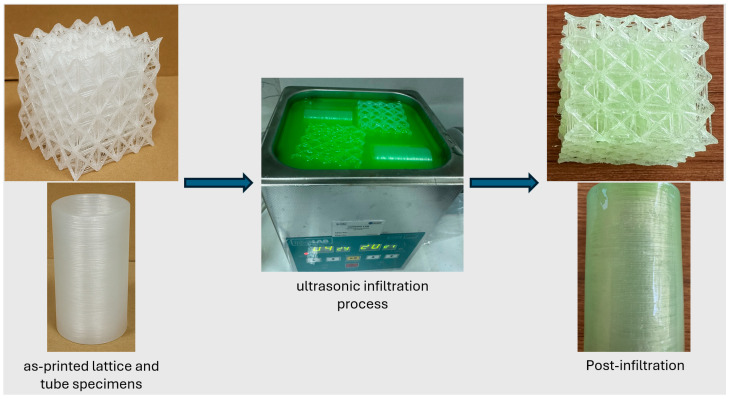
Epoxy infiltration process applied to 3D-printed PP specimens.

Lattice and tube structures were subjected to epoxy infiltration to strengthen interlayer bonds and fill microvoids formed during production, thereby improving overall structural integrity. The infiltration process was conducted in an ISO-LAB ultrasonic bath (1.3 L capacity, 40 kHz). Before infiltration, the epoxy was preconditioned to the desired temperature to stabilize the viscosity at the target level, ensuring consistent test conditions across all specimens. The samples were then fully immersed in the epoxy for approximately 120 s until all air bubbles dissipated, while ultrasonic agitation promoted uniform penetration into the intricate lattice and tube structures. During this period, the temperature increased by an average of 3 °C, but this change remained within a controlled range that improved flowability without exceeding the targeted viscosity window. Preliminary tests showed that epoxy viscosity decreased markedly up to ~36 °C and continued to drop to ~126 cP at 60 °C, which promoted surface run-off and reduced pore retention. Accordingly, the infiltration temperature was capped at 36 °C (150–180 cP) to balance penetration with retention and to avoid surface run-off at very low viscosity values. The infiltration steps are schematically illustrated in Figure 2.

### 2.3. Experimental Matrix and Testing Protocol

The experimental design was based on two main variables: structural geometry (lattice and tube) and epoxy viscosity. Four viscosity levels were selected (150–180 cP, 300–350 cP, 500–560 cP, and 1000–1070 cP) to represent the full range of flowability, from low to high, observed in the epoxy system within the tested temperature window. At each viscosity level, both lattice and tube samples were produced and infiltrated (Table 2). Sample groups were designated with the letter “L” for lattice and “T” for tube, while viscosity levels were coded as V1–V4. Control samples without infiltration were labeled L-C and T-C. Due to their 45° printing orientation, lattice structures tend to exhibit layer stacking and void formation, whereas tube structures form more homogeneous surfaces through vertical layer stacking. A primary objective of this study was to evaluate how these geometric differences influence epoxy adhesion and the resulting energy absorption capacity.

The energy absorption behavior of the samples was assessed using Energy Absorption (EA), Specific Energy Absorption (SEA), and Mean Crushing Force (MCF) parameters. Additionally, the post-infiltration mass increase (Δm) was measured as an indicator of the epoxy’s ability to fill internal voids and its adhesion efficiency, both of which are viscosity-dependent. Three replicates were tested for each group, and the results were analyzed using correlation methods.

### 2.4. Characterization Techniques

The infiltration efficiency, microstructural filling, and energy absorption performance of the specimens were investigated through mechanical, physical, and microstructural analyses. These included mass and density measurements, scanning electron microscopy (SEM), and visual penetration observations using pigmented epoxy. Mechanical behavior was examined via quasi-static compression tests.

#### 2.4.1. Mass Measurements

Sample masses before and after epoxy infiltration were measured using an analytical balance (Isolab GmbH, Wertheim, Germany) with a precision of ±0.0001 g. Three measurements were taken for each sample, and the average values were calculated. The mass difference (Δm) was obtained by subtracting the initial mass from the final mass and was used as an indicator of epoxy retention. These data were incorporated into both the infiltration efficiency assessment and SEA calculations. In addition, the CAD-based theoretical mass, the as-printed mass, and the post-infiltration mass were compared to estimate porosity reduction. The relative mass uptake (Δm%) between these states was interpreted as an indirect but reliable measure of infiltration efficiency.

#### 2.4.2. SEM and Visual Penetration

Surface morphology and the microstructural effects of epoxy infiltration were examined using a scanning electron microscope (ZEISS, Oberkochen, Germany) and a digital microscope (Insize ISM-PM2005A, Suzhou, China). SEM images were acquired at 40× magnification for overall morphology and at 200× for microvoid distribution and size analysis. Prior to imaging, all samples were coated with a gold–palladium layer to ensure conductivity. The extent of pore filling was assessed by combining SEM and mass uptake results, while the pigmented epoxy enabled direct visual confirmation of infiltration depth under the digital microscope. Cross-sectional SEM images provided evidence of whether mass gain originated from internal filling or from surface film formation. Such cross-sections were readily obtained for tube specimens, but due to the brittle nature of cured epoxy, reliable sections for lattice structures could not be prepared; infiltration in lattices was therefore confirmed indirectly.

#### 2.4.3. Quasi-Static Compression Testing

**Figure 3:**
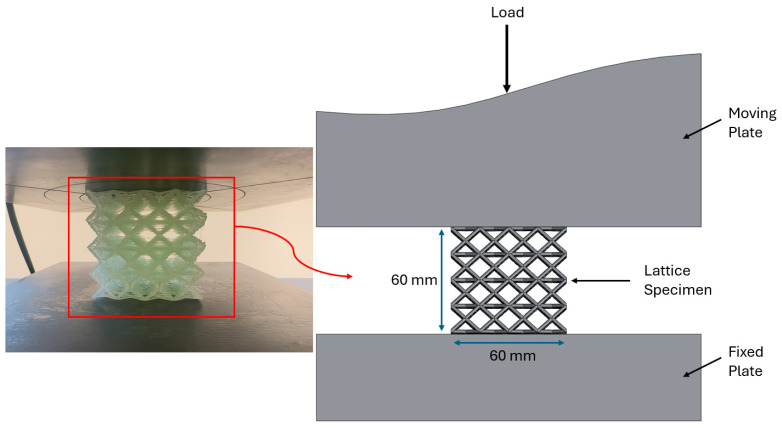
Quasi-static compression test setup for lattice specimen.

Quasi-static compression tests were conducted on a 200 kN universal testing machine (DOTEK) at room temperature, with a constant crosshead speed of 2 mm/min applied parallel to the sample axis. Force–displacement data were recorded continuously, and EA, SEA, and MCF parameters were calculated from these data. The test setup and loading configuration for a lattice specimen are shown in Figure 3.

### 2.5. Crashworthiness Criteria

The energy absorption capacity of crashworthy components is typically evaluated using several key mechanical indicators. In this study, the crashworthiness of the samples was examined using total energy absorption (*EA*), specific energy absorption (SEA), and mean crushing force (MCF) values. These parameters were derived from the load–displacement curves obtained during compression tests.

The EA value, which is obtained by integrating the area under this curve, is given in Equation (1).

(1) $$EA=\int_{x=0}^{x=l}F\left(x\right)dx$$

Here, *l* denotes the total deformation distance, and *F(x)* is the compressive force measured during the test. Specific Energy Absorption (SEA), defined as the energy absorbed per unit mass, is calculated using Equation (2).

(2) SEA=EA/m 

Here, *m* is the mass of the sample. Mean Crushing Force (MCF) represents the average of the forces measured throughout the deformation and is defined by Equation (3).

(3) $$MCF=\frac{1}{l}\int_{x=0}^{x=l}F\left(x\right)dx$$

High *SEA* and *MCF* values reflect enhanced energy absorption capacity and greater deformation efficiency.

## 3. Results and Discussion

This section investigates the morphological, mechanical, and mass-based changes observed in 3D-printed polypropylene (PP) lattice and cylindrical tube structures following epoxy infiltration. The study evaluates the effects of epoxy viscosities ranging from 150 to 1070 cP on pore volume fraction, mass increase (Δm), and energy absorption capacity. These findings were analyzed using morphological examinations conducted before and after infiltration, scanning electron microscopy (SEM), digital microscopy (DM), and quasi-static compression tests yielding EA, SEA, and MCF parameters. The results showed that viscosity and geometric configuration (lattice and cylindrical tube) played a critical role in infiltration efficiency, directly influencing mechanical performance.

### 3.1. Morphological Analysis

The morphological effects of epoxy infiltration were examined in detail using DM and SEM analyses. Variations in viscosity were found to affect surface film formation, pore filling ratio, and interlayer bonding behavior.

#### 3.1.1. Morphological Analysis of Tube Structures

**Figure 4:**
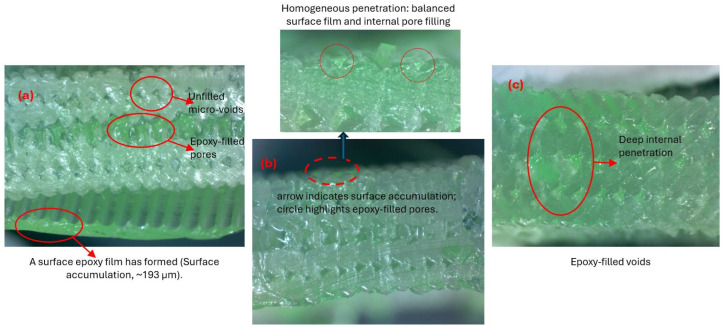
Viscosity-dependent epoxy infiltration and surface retention in tube structures. (**a**) T-V4: 1000–1070 cP, (**b**) T-V2: 300–350 cP, (**c**) T-V1: 150–180 cP.

**Figure 5:**
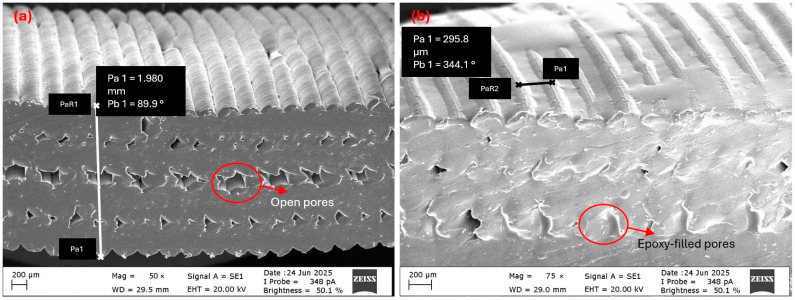
SEM images of the T-V2 sample before (**a**) and after (**b**) infiltration.

**Figure 6:**
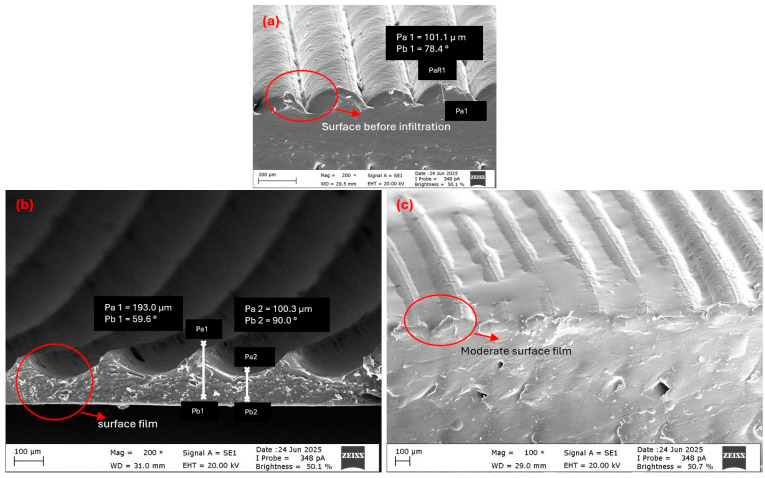
SEM images of tube surfaces before and after infiltration: (**a**) PP tube surface (before infiltration), (**b**) surface shell after high-viscosity epoxy (T-V4, 1000–1070 cP), (**c**) surface after medium-viscosity epoxy (T-V2, 300–350 cP).

Morphological analysis revealed that epoxy viscosity strongly influenced both sur-face film formation and internal pore filling in tube structures. Cross-sectional images confirmed that the green-pigmented epoxy successfully penetrated the inner regions and spread uniformly throughout the tube structure, especially at lower viscosities. In the high-viscosity T-V4 sample (Figure 4a), limited fluidity restricted penetration, causing epoxy to accumulate in large pores and form a dense outer surface shell up to 193 µm thick. Similar pore morphologies prior to infiltration are visible in (Figure 5a), while SEM observations further confirmed the thick outer layer in (Figure 6b). This surface film filled micro-indentations, reduced roughness, and created a barrier at layer interfaces. The green surface shell visible in Figure 4a, formed by intense surface adhesion, was carefully re-moved to enable clearer visualization of the underlying structure.

In contrast, the low-viscosity T-V1 sample (Figure 4c) exhibited deep penetration due to high fluidity, substantially increasing the fill ratio. However, a thick crust did not form; instead, epoxy adhesion was localized mainly in micro-pores, indicating that low viscosity promoted effective wetting without significant surface buildup.

The medium-viscosity T-V2 sample (Figure 4b and Figure 5b) demonstrated the most balanced behavior. Irregular pores visible prior to infiltration (Figure 5a) were effectively filled, creating a more homogeneous matrix between pore walls. On the surface, a thinner and more dispersed film formed compared to the T-V4 sample, with the natural printing texture partially preserved (Figure 6c). Adhesion was concentrated in indentations along 3D printing traces, highlighting a dual mechanism of pore filling and controlled surface bonding.

These findings confirm that viscosity is decisive for controlling infiltration in tube structures. In tube structures, T-V4 favored surface adhesion, T-V1 enabled deep penetration, and T-V2 provided the best balance, as evidenced in Figure 4, Figure 5 and Figure 6.

#### 3.1.2. Morphological Analysis of Lattice Structures

**Figure 7:**
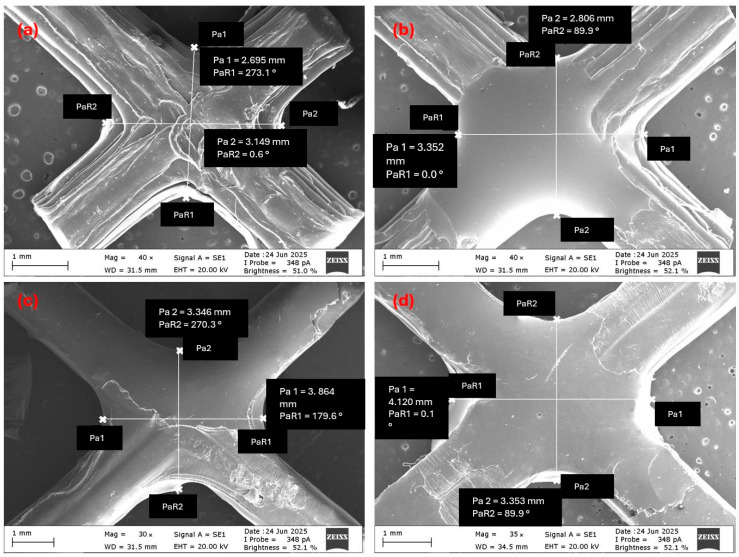
Morphological changes in lattice surfaces under different infiltration parameters: (**a**) lattice strut with thin epoxy film, (**b**) lattice surface with partial filling, (**c**) lattice with dense surface crust, and (**d**) balanced infiltration with moderate film thickness.

The complex geometry of lattice structures, characterized by multiple printing angles and short arm segments, led to pronounced surface roughness. During FDM printing, frequent nozzle direction changes and sharp layer transitions generated micro-indentations and ripples on the struts (Figure 7a). These morphological features are highlighted in Figure 7a, where they are visible prior to infiltration and play a key role in subsequent epoxy wetting. These morphological features strongly influenced epoxy infiltration by controlling film formation and surface adhesion.

SEM observations confirmed that low-viscosity epoxy (L-V1, 150–180 cP; Figure 7b) formed a thin and homogeneous film layer across the surface. Its high fluidity enabled effective wetting of surface irregularities, but the resulting surface crust thickness remained limited. This indicates that low viscosity favored penetration without excessive film buildup.

Accordingly, low viscosity enabled penetration without excessive film buildup. Figure 7b shows a thin uniform film that preserved the strut texture, confirming minimal accumulation. Consistent SEM, DM, and Δm% results further showed that infiltration extended beyond the surface layer into micro-indentations and inner pores.

In contrast, high-viscosity epoxy (L-V4, 1000–1070 cP; Figure 7d) exhibited poor internal penetration due to its restricted flowability. Instead, epoxy accumulated locally in the surface roughness, creating a thick surface shell. SEM images revealed localized clusters of epoxy, which enhanced surface adhesion but significantly constrained infiltration into inner pores. This is evident in Figure 7d, where a dense surface crust is observed, indicating restricted flow into the inner pores and the dominance of surface accumulation over internal filling.

The medium-viscosity epoxy (L-V2, 300–350 cP; Figure 7c) achieved a more favorable balance between surface energy and flowability. SEM analysis showed that epoxy infiltrated micro-indentations within the printed layers, forming weld-like bonds that strengthened the interlayer interfaces. At the same time, only a moderate film was observed on the surface, preserving the texture of the printed struts. As annotated in Figure 7c, epoxy infiltrated micro-indentations while forming only a moderate surface film, thereby reinforcing the interlayer bonds while preventing the formation of a thick external shell as observed in L-V4.

Overall, the comparative analysis (Figure 7b–d) indicates that epoxy viscosity is a key factor controlling infiltration in lattice structures. High viscosity promoted surface adhesion, low viscosity enabled penetration with minimal film formation, and medium viscosity provided the best balance. The differences illustrated in Figure 7b–d, with annotations highlighting thin films (Figure 7b), thick crusts (Figure 7d), and balanced infiltration (Figure 7c), support the conclusion that medium viscosity offers the most effective infiltration regime. Consistent with the tube results, medium-viscosity epoxy (L-V2) was therefore identified as the optimal condition for achieving both uniform internal filling and controlled surface film thickness.

### 3.2. Mass Gain and Infiltration Effectiveness

The mass increase (Δm) following epoxy infiltration reflects both the extent of internal pore filling and the contribution of the surface film. As detailed in Table 3, mass values were determined from three references: CAD mass (theoretical, void-free mass), as-printed mass (porous structure directly after 3D printing), and post-infiltration mass (final state after epoxy infiltration). The Mass Uptake (%) ratio, derived from these values, quantifies infiltration effectiveness but requires careful interpretation, as epoxy viscosity simultaneously governs pore penetration and surface film buildup. Therefore, Δm% values were carefully interpreted in conjunction with morphological evidence to avoid overestimating porosity reduction due to surface accumulation.

Mass Uptake (%) expresses the percentage of mass change after infiltration and was calculated using the following formula:

(4) $$Mass\;Uptake\;(\%)=\frac{m_{infiltrated}-m_{as-printed}}{m_{as-printed}}\times 100$$

At low viscosities (150–180 cP), epoxy exhibited high flowability, penetrating the internal pores effectively while forming only a thin surface film. In contrast, high viscosities (1000–1070 cP) restricted penetration, resulting in a dense surface shell that reached up to 193 µm in thickness (SEM, Figure 6b). In these cases, the measured mass increase was dominated by surface accumulation rather than internal filling, meaning high Mass Uptake (%) values did not necessarily indicate porosity reduction. Thus, mass data should always be evaluated in conjunction with morphological analysis.

For tubular structures, the trend of increasing mass with higher viscosity was evident, though the underlying mechanisms differed. Low-viscosity tubes showed limited surface film formation and, consequently, smaller mass increases. In the medium-viscosity range (300–560 cP), infiltration balanced both internal penetration and surface adhesion. While T-V2 and T-V3 samples exhibited similar mass gains, SEM analysis revealed different mechanisms: T-V2 showed deeper internal penetration, whereas T-V3 developed a thicker surface film.

In lattice structures, infiltration was complicated by angular geometry, high pore volume, and pronounced surface roughness. These features promoted epoxy accumulation on the surface, particularly at medium and high viscosities. As a result, elevated Mass Uptake (%) values were often associated with surface films rather than pore filling. The L-V2 sample (300–350 cP) best represented balanced infiltration, combining homogeneous penetration with limited surface film formation.

Overall, both numerical data and SEM analyses confirm that medium-viscosity samples (T-V2 and L-V2) achieved the most balanced infiltration performance. Their high internal density, uniform film distribution, and controlled mass gain identify the medium-viscosity range as the optimal condition for epoxy infiltration in both structural types. It is noteworthy that a mass uptake of approximately 37% (for L-V2) corresponds to a balanced state of internal filling and surface film formation, as confirmed by Table 3 and morphological analyses (Figure 7c). This value reflects effective penetration into micro-pores without excessive surface accumulation, thereby indicating optimal infiltration efficiency. This efficiency is directly associated with the enhanced SEA values observed in medium-viscosity specimens.

### 3.3. Mechanical Behavior Under Compression

**Figure 8:**
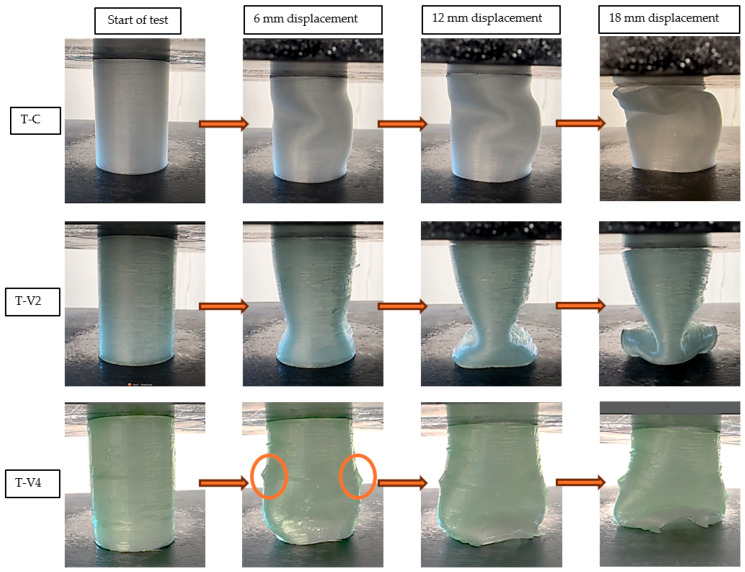
Comparative deformation behavior of tube specimens (T-C, T-V2, T-V4) under compression at selected displacement levels. Circles in T-V4 highlight localized crack initiation regions.

**Figure 9:**
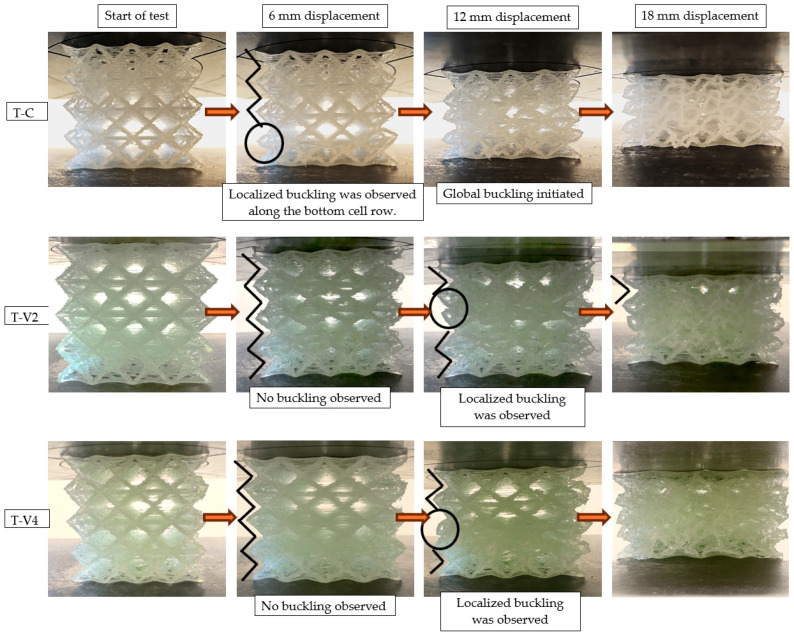
Compression-induced deformation of lattice specimens (L-C, L-V2, L-V4) at displacement levels of 0, 6, 12, and 18 mm. Circles highlight localized buckling zones, and arrows indicate the progression of global buckling.

**Figure 10:**
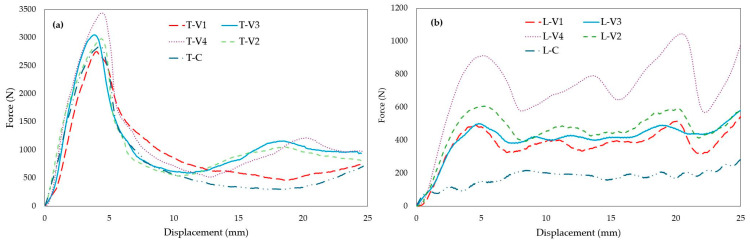
Force–displacement curves: (**a**) Tube specimens, (**b**) Lattice specimens (viscosity-based comparison.

**Figure 11:**
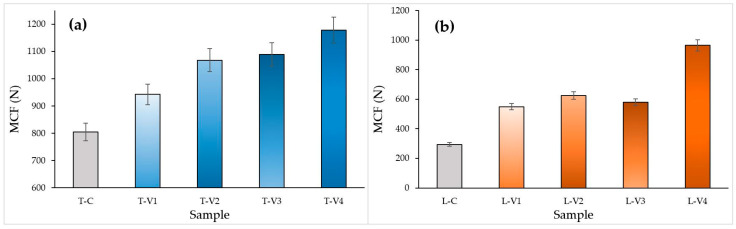
MCF values at different epoxy viscosity levels: (**a**) Tube structures, (**b**) Lattice structures. Blue bars represent tube specimens, orange bars represent lattice specimens, and grey bars represent control specimens.

**Figure 12:**
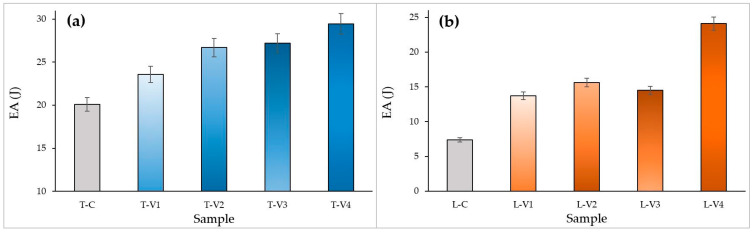
EA MCF values at different epoxy viscosity levels: (**a**) Tube structures, (**b**) Lattice structures. Blue bars represent tube specimens, orange bars represent lattice specimens, and grey bars represent control specimens.

**Figure 13:**
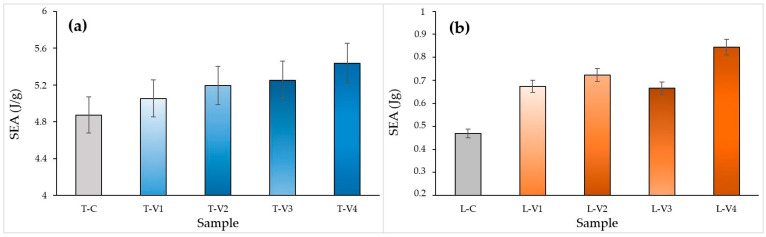
SEA values at different epoxy viscosity levels: (**a**) Tube structures, (**b**) Lattice structures. Blue bars represent tube specimens, orange bars represent lattice specimens, and grey bars represent control specimens.

Mechanical test results demonstrated that epoxy infiltration enhanced energy absorption (EA), specific energy absorption (SEA), and maximum compressive force (MCF) in both lattice and tube structures. The nature and underlying mechanisms of these improvements, however, varied depending on specimen geometry. Visual analyses of specimen deformation (Figure 8 for tubes and Figure 9 for lattices) qualitatively revealed the influence of epoxy infiltration on structural integrity and buckling mechanisms. The primary evaluation relied on force–displacement curves (Figure 10), with complementary parameters (EA, SEA, and MCF; Figure 11, Figure 12 and Figure 13) providing further validation. Since lattice and tube structures inherently differ in mass distribution and deformation kinematics, EA values were not directly compared across geometries. Instead, SEA (EA/m) was used to evaluate structural efficiency on a per-mass basis, providing a fair comparison between different configurations. All tests were performed at a consistent strain rate (0.001 s−1) to ensure that infiltration efficiency could be assessed independently of geometry-related effects.

In Figure 8, the effects of different epoxy viscosities on the deformation behavior of tube structures are clearly visible. The epoxy-free control specimen (T-C) exhibited asymmetric buckling under increasing load due to manufacturing-induced layer separations as well as weak interfacial bonding. In contrast, the medium-viscosity specimen (T-V2) delayed deformation and maintained its structural form for a longer duration. SEM observations and mass uptake data confirmed that this improvement was due to effective void filling, which enhanced load distribution. On the other hand, the high-viscosity specimen (T-V4) developed extensive surface delaminations as deformation progressed. These separations were likely caused by insufficient epoxy penetration into the interior, the limited interfacial bonding between polypropylene and epoxy, and the formation of a thick surface shell. Overall, the results indicate that epoxy viscosity is a decisive parameter affecting not only internal filling efficiency but also the deformation mode and structural integrity.

The deformation behavior of lattice structures under compression (Figure 9) revealed the influence of epoxy viscosity more distinctly. The epoxy-free control specimen (L-C) exhibited local buckling in the bottom row of cells at a displacement of 6 mm, which then progressively spread to all cells as deformation advanced. This behavior confirmed that manufacturing-induced voids and weak interlayer bonds led to structural instability. Conversely, in the L-V2 and L-V4 specimens, buckling remained confined to a single row of cells up to a displacement of 12 mm, while the overall structural form was preserved. This improvement is attributed to the ability of epoxy infiltration to enhance cell stability. In the later stages of testing, the L-V2 specimen achieved an optimal balance between viscosity and filling ratio, and was the specimen that sustained the load for the longest duration.

Compression tests also highlighted fracture-related mechanisms. SEM analyses revealed that in high-viscosity specimens (T-V4 and L-V4), cracks typically initiated at stress concentrations within the brittle epoxy-rich surface film and then propagated along weak epoxy–polypropylene interfaces, leading to premature delamination. In contrast, medium-viscosity specimens (T-V2 and L-V2) exhibited delayed crack initiation and more tortuous crack paths, as epoxy penetration into micro-indentations promoted mechanical interlocking and stronger adhesion. This improved bonding redistributed stresses more evenly, thereby reducing the likelihood of interfacial debonding and catastrophic failure.

#### 3.3.1. Maximum Compressive Force (MCF) and Force-Displacement Behavior

The pronounced effect of epoxy on mechanical performance was evaluated through the force–displacement curves (Figure 10) and corresponding MCF values (Figure 11). MCF is defined as the peak load attained before the onset of initial buckling.

In tube structures, MCF values gradually increased with rising viscosity. The high-viscosity specimen T-V4 reached the highest peak load, approximately 3100 N, due to the resistance provided by the epoxy-rich surface layer during the initial stages of deformation. However, as deformation progressed, cracking of the shell occurred, and a lack of sufficient internal reinforcement caused a rapid decline in load-bearing capacity. In comparison, the medium-viscosity specimens T-V2 and T-V3 reached similar peak loads, but their internal void infiltration balanced load transfer and enabled a more stable load-bearing response throughout deformation. For the low-viscosity specimen T-V1, insufficient epoxy bonding led to early stiffness loss and reduced load capacity.

In lattice structures, the influence of viscosity on mechanical behavior was more pronounced due to their complex cellular geometry. Mechanical tests (Figure 11b) confirmed that the high-viscosity specimen L-V4 achieved the highest MCF value (964 N) as the thick surface film provided additional resistance against buckling. This improvement, however, came at the expense of limited epoxy penetration, which concentrated stresses at weak interfaces and caused sudden load drops during deformation. In the medium-viscosity specimen L-V2, more efficient filling of internal voids resulted in a more even stress distribution, enabling the structure to sustain higher loads for a longer duration and yielding a high MCF value. By contrast, the low-viscosity specimen L-V1 and the control specimen L-C lost structural integrity prematurely and exhibited low load-bearing capacity.

#### 3.3.2. Energy Absorption Capacity (EA and SEA)

The influence of epoxy infiltration on energy absorption (EA) and specific energy absorption (SEA) was comparatively evaluated for both structural types using the data in Figure 12 and Figure 13.

In tube structures, EA exhibited a gradual increase with rising viscosity, from 20.11 J in the control specimen to 29.45 J at the highest viscosity level (Figure 12a). The high-viscosity specimen T-V4 achieved the highest EA, approximately 30 J, owing to the contribution of the thick epoxy surface film to energy dissipation during deformation. Specimens T-V2 and T-V3 also reached comparable values and displayed a more stable energy absorption profile. This was attributed to deeper void penetration. SEA values, however, remained relatively close, indicating that the observed increase was primarily associated with mass gain (Figure 13a).

In lattice structures, the effect of viscosity on EA and SEA was more pronounced (Figure 12b and Figure 13b). The high-viscosity specimen L-V4, benefiting from the additional buckling resistance provided by the thick epoxy surface film, demonstrated the highest performance, reaching an SEA of 0.84 J/g (approximately 80% higher than the control specimen). This enhancement occurred because the thick surface film temporarily delayed global buckling but restricted the redistribution of stresses once cracking initiated, leading to sudden load drops. In contrast, the medium-viscosity specimen L-V2 achieved a more uniform distribution of epoxy, which reinforced weak interlayer bonds and enabled more balanced energy absorption. These findings indicate that, in lattice structures, epoxy acts not only as a filler but also as a structural stabilizer that governs energy distribution.

#### 3.3.3. General Discussion

The mechanical test results demonstrated that epoxy infiltration generally enhanced the performance of both structural types. However, the underlying mechanisms of these improvements varied according to structural geometry. In tube structures, the influence of viscosity was more evident in parameters such as MCF and EA, whereas in lattice structures, more substantial improvements were observed in specific parameters such as SEA. This difference is closely related to the critical contribution of surface film thickness to the performance of lattice structures, which are prone to buckling.

Within the medium-viscosity range (T-V2 and T-V3 specimens), a balanced combination of internal penetration and surface adhesion was achieved. Consequently, specimens T-V2 and L-V2 indicate the optimal viscosity range for both structural types in terms of infiltration efficiency and mechanical performance balance.

### 3.4. Correlation Analysis and Infiltration Performance

The effect of epoxy infiltration on structural performance was assessed through a multifaceted approach that integrated mechanical testing, morphological observations, and mass uptake data. Pearson correlation analyses demonstrated statistically significant linear relationships among viscosity, mass gain (Δm), maximum compressive force (MCF), total energy absorption (EA), and specific energy absorption (SEA) in both lattice and tube configurations. Although the strength of these relationships varied with structural geometry, they were directly linked to the viscosity-dependent infiltration behavior of the epoxy.

**Figure 14:**
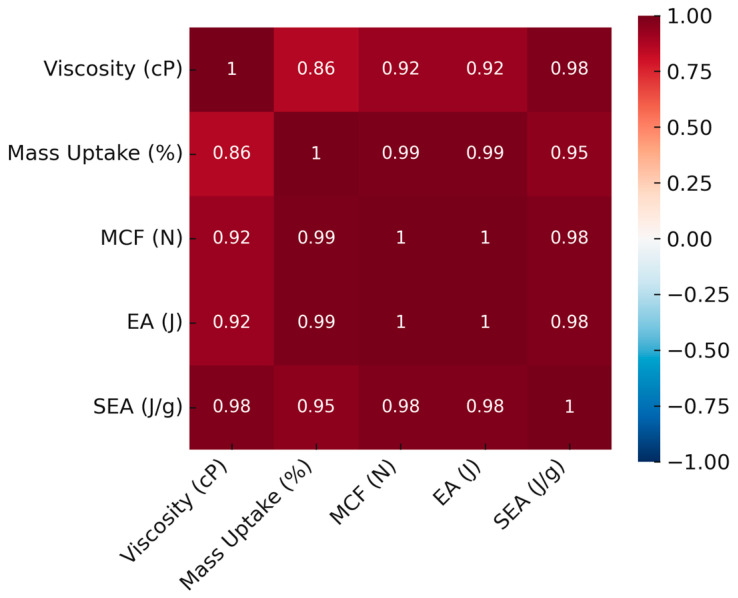
Heatmap of correlations for tube structures.

In tube structures (Figure 14), the correlation analysis revealed that increasing viscosity led to notable rises in both mass retention and mechanical performance. Strong positive correlations were observed between viscosity and SEA (r = 0.98), EA (r = 0.92), and MCF (r = 0.92), while Δm showed near-perfect correlations with both EA and MCF (r = 0.99). These findings underscore the direct and robust connection between infiltration efficiency and mechanical response.

Morphologically, the high-viscosity specimen T-V4 formed a thick epoxy surface film but exhibited limited internal penetration (Figure 4a and Figure 6). Nevertheless, it achieved the highest EA and MCF values, indicating that the surface film substantially contributed to load distribution and mechanical enhancement. However, the restricted epoxy content in the core regions may have weakened interfacial bonds.

In contrast, the medium-viscosity specimen T-V2 presented a more balanced infiltration profile between viscosity and internal filling. It achieved a high MCF (897 N) despite a relatively modest mass gain (37.88%) (Figure 4b and Figure 7c).

The correlations in lattice structures followed similar overall trends to those in tube structures, yet the greater geometric complexity required a more delicate balance. The relatively lower correlation between viscosity and SEA (r = 0.85) indicates that epoxy adherence within the lattice geometry was limited and infiltration was less uniform. By contrast, strong correlations between Δm and both MCF and EA (r = 0.99) demonstrated that filling efficiency directly governed mechanical performance. Medium-viscosity specimens L-V2 and L-V3, with approximately 37% mass uptake, achieved high SEA (0.84 J/g) and EA (252 J) values. L-V2 displayed a well-balanced combination of internal filling and surface film formation, yielding high mechanical performance despite lower mass gain. In L-V4, higher viscosity produced a thick surface film but restricted internal penetration, leading to localized stress concentrations and premature failure during deformation (Figure 9)

**Figure 15:**
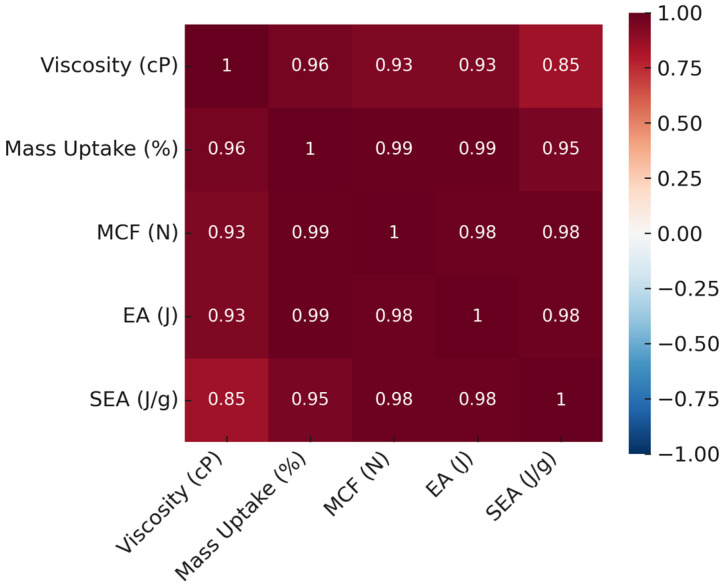
Heatmap of correlations for lattice structures.

For both structural types, improvements in mechanical performance were governed not only by viscosity but also by infiltration efficiency, film distribution, and internal filling. High correlation coefficients, when interpreted alongside morphological integrity (Figure 15), highlight T-V2 and L-V2 as optimal specimens in terms of performance balance. The results clearly demonstrate that energy absorption and load-bearing capacity depend not only on the extent of filling but also on multiple interrelated parameters, including the penetration pattern of the epoxy, film thickness, and micropore filling. Within this framework, medium-viscosity epoxies in the range of 300–560 cP provided the most balanced infiltration–performance relationship for both lattice and tube configurations.

## 4. Conclusions

This study demonstrated that the mechanical performance of polypropylene-based lattice and tube structures fabricated via FDM 3D printing can be significantly enhanced through viscosity-controlled epoxy infiltration. The findings revealed that while viscosity alone cannot fully explain the performance outcomes, an optimal viscosity range—when combined with efficient infiltration—proved decisive in improving both energy dissipation and load-bearing capacity. In particular, medium-viscosity epoxies in the range of 300–560 cP provided the highest structural integrity by achieving a balanced synergy between internal filling and surface film thickness.

Correlation and morphological analyses reinforced these statistical results. High-viscosity epoxies formed thick surface films but exhibited limited penetration into the core regions, whereas low-viscosity epoxies penetrated deeply but left weak interlayer bonds. In contrast, the efficient infiltration achieved with medium-viscosity epoxy in specimens T-V2 and L-V2 resulted in high mechanical performance despite relatively low mass gain. Taken together, numerical data and SEM analyses confirm that T-V2 and L-V2 achieved the most balanced infiltration performance. These outcomes underscore the interplay between viscosity and structural geometry as a critical design parameter for porous 3D-printed structures.

The infiltration-based strengthening strategy developed in this study offers an innovative alternative to existing methods in the literature. 3D-printed structures, which typically exhibit limited mechanical strength due to interlayer bond weaknesses and manufacturing-induced voids, can overcome these limitations and achieve high performance through this approach. The proposed infiltration mechanism may broaden the application range of 3D-printed materials—particularly in critical sectors such as aerospace, defense, and automotives—for use in load-bearing components and crash energy-absorbing structures.

The findings of this study also provide practical design guidelines. For crashworthy components in the automotive and defense sectors, where maximizing energy absorption and stability under high loads is essential, medium-to-high viscosity epoxies (e.g., T-V3 and L-V4) are recommended due to their ability to form thicker surface films and enhance crash energy dissipation. Conversely, for lightweight aerospace and robotics applications, where minimizing mass gain is critical, lower-viscosity epoxies (e.g., T-V2 and L-V2) are preferable, as they enable effective internal reinforcement while maintaining high SEA values with reduced weight penalties. Thus, infiltration viscosity can be tailored to meet diverse engineering requirements, linking the experimental findings of this study directly to real-world design strategies.

## 5. Future Work

This study demonstrates how viscosity-controlled epoxy infiltration improves the mechanical performance of 3D-printed structures, yet it also raises new questions for future research. Key areas for further investigation include:

Repeated Infiltration Mechanisms: The effects of multi-stage infiltration using low-viscosity epoxy should be examined to assess improvements in structural performance and pore filling. This may offer a new balance between process complexity and mechanical gain.

Advanced Imaging Techniques: Techniques such as micro-CT are critical for quantitative evaluation of internal morphology and infiltration depth, enabling a more detailed assessment of structural integrity and filling homogeneity.

Different Materials and Geometries: Future studies should explore different polymers (e.g., PLA, ABS, composites) and various lattice geometries (e.g., octahedron, cube) to test the adaptability of the proposed infiltration method across applications.

Scalability: The scalability of sonication-assisted infiltration must be addressed for industrial use. Research should investigate large-scale ultrasonic baths or localized ultrasonic probes to determine the feasibility of applying the technique to larger components.

Long-Term Durability: The durability of epoxy-infiltrated PP structures under environmental conditions (humidity, thermal cycling, UV exposure) and under cyclic or fatigue loading should be studied to validate industrial applicability.

## Figures and Tables

**Table 1:** 3D printing parameters for PP specimens.

Parameter	Value
Printer model	ekser plus
Nozzle diameter	0.4 mm
Layer height	0.2 mm
Nozzle temperature	240 °C
Bed temperature	80 °C
Printing orientation	Vertical (Z-axis)
Printing speed	40 mm/s
B-5	5
Infill density	%100 (solid)
Filament diameter	1.75 mm

**Table 2:** Specimen groups with different epoxy viscosities.

Code	Geometry	İnfiltration Epoxy Viscosity (cP)
L-C	Lattice	--
L-V1	Lattice	150–180
L-V2	Lattice	300–350
L-V3	Lattice	500–560
L-V4	Lattice	1000–1070
T-C	Tube	--
T-V1	Tube	150–180
T-V2	Tube	300–350
T-V3	Tube	500–560
T-V4	Tube	1000–1070

**Table 3:** Mass uptake ratios of lattice and tube specimens infiltrated with different viscosity epoxy systems.

Specimen	CAD Mass (g)	As-Printed Mass (g)	Post-Infiltration Mass (g)	Mass Uptake (%)
L-V1	19.597	15.684	20.415	30.164
L-V2	19.597	15.684	21.624	37.872
L-V3	19.597	15.684	21.806	39.033
L-V4	19.597	15.684	28.543	81.988
T-V1	4.559	4.126	4.663	13.015
T-V2	4.559	4.126	5.143	24.648
T-V3	4.559	4.126	5.186	25.690
T-V4	4.559	4.126	5.419	31.337

## Data Availability

The original contributions presented in this study are included in the article. Further inquiries can be directed to the corresponding author.
